# Supplementary figures and images for: Suicidal ideation, plan, and attempt among men who have sex with men in Nepal: Findings from a cross-sectional study
Source: PLOS Glob Public Health. 2023 Nov 22;3(11):e0002348. doi: 10.1371/journal.pgph.0002348 (PMC10664887; doi:10.1371/journal.pgph.0002348)

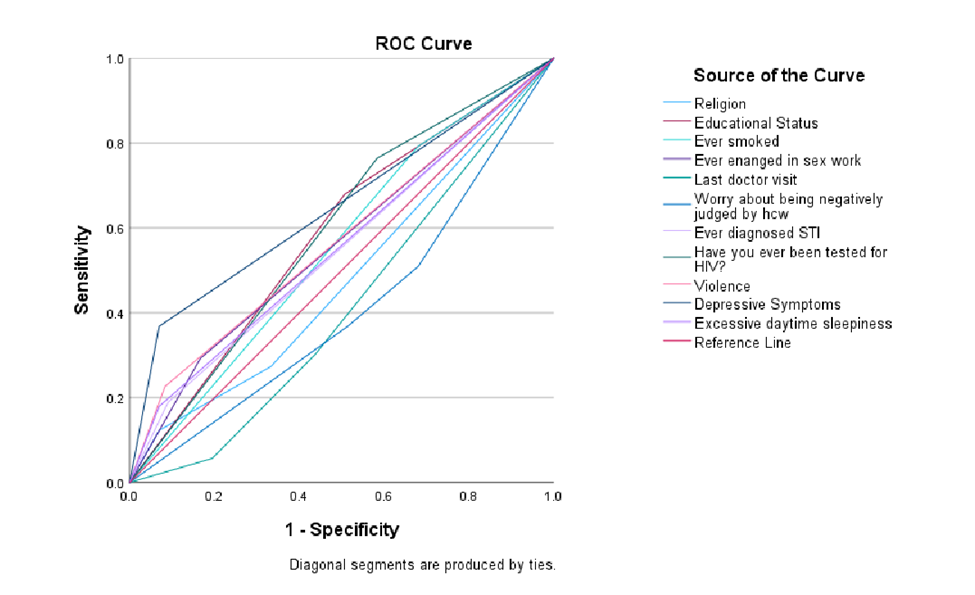

Supplement: S1 Fig — (TIFF) [file pgph.0002348.s001.tiff]

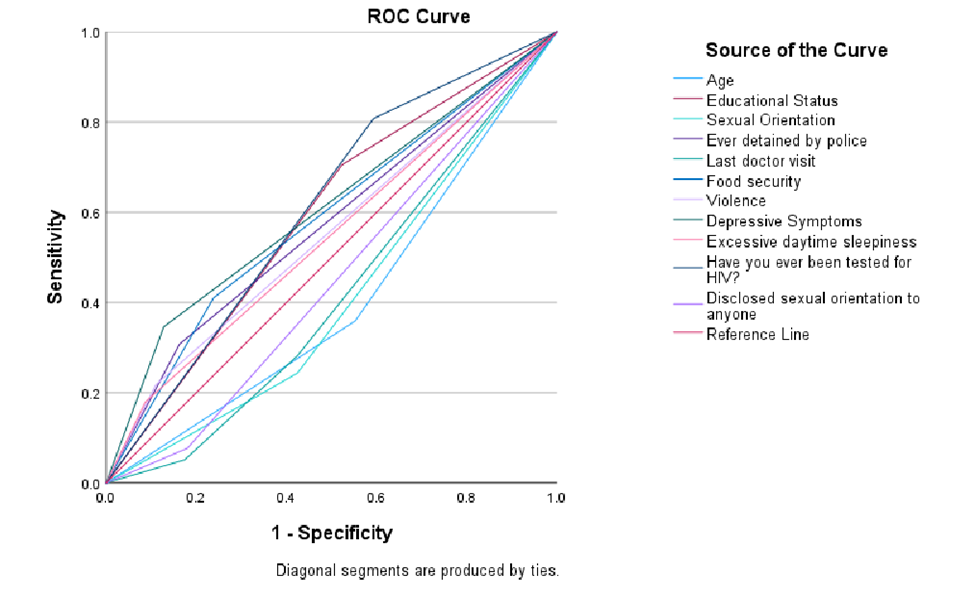

Supplement: S2 Fig — (TIFF) [file pgph.0002348.s002.tiff]

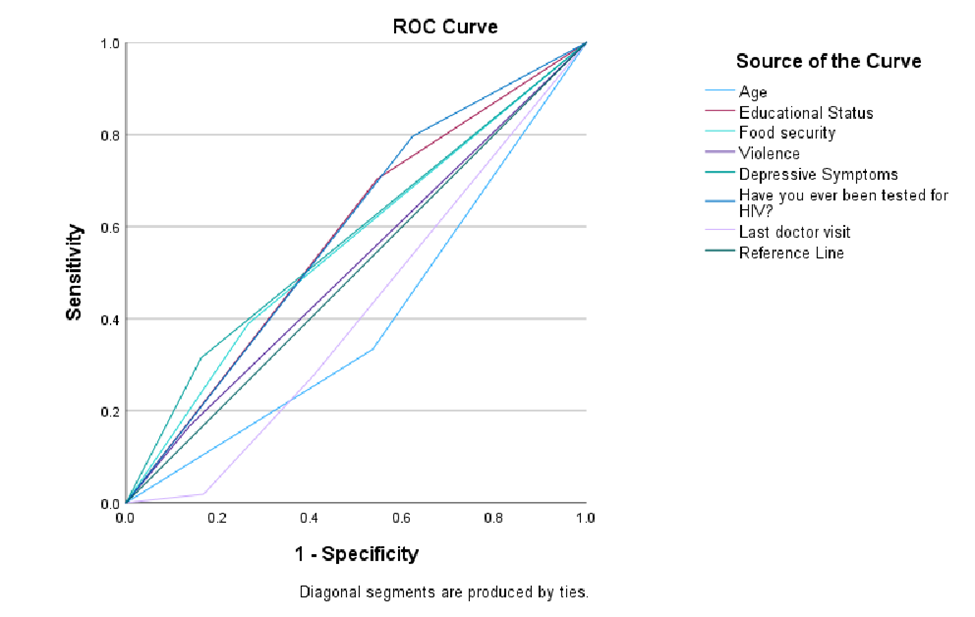

Supplement: S3 Fig — (TIFF) [file pgph.0002348.s003.tiff]
