# Supplementary material for: Suicidal ideation, plan, and attempt among men who have sex with men in Nepal: Findings from a cross-sectional study
Source: PLOS Glob Public Health. 2023 Nov 22;3(11):e0002348. doi: 10.1371/journal.pgph.0002348 (PMC10664887; doi:10.1371/journal.pgph.0002348)
Supplement: S1 Table — (DOCX) [file pgph.0002348.s004.docx]

**S1 Table:** Distribution of area under the curve of the suicidal ideation with independent variables

| **Test Result Variable(s)** | **Area** | **Std. Error^a^** | **Asymptotic Sig.^b^** | **Asymptotic 95% Confidence Interval** | |
| --- | --- | --- | --- | --- | --- |
|  |  |  |  | **Lower Bound** | **Upper Bound** |
| Religion | .481 | .037 | .611 | .408 | .554 |
| Educational status | .586 | .036 | .018 | .515 | .657 |
| Ever smoked | .556 | .036 | .125 | .484 | .627 |
| Ever engaged in sex work | .563 | .037 | .090 | .490 | .636 |
| Last time doctor visit | .415 | .036 | .018 | .345 | .485 |
| Worry about being negatively judged by health care workers | .409 | .037 | .012 | .337 | .480 |
| Ever diagnosed with STI | .549 | .037 | .186 | .476 | .622 |
| Ever tested HIV | .590 | .036 | .012 | .520 | .661 |
| Violence | .572 | .037 | .054 | .499 | .644 |
| Depressive symptoms | .649 | .036 | .000 | .578 | .720 |
| Daytime sleepiness | .555 | .037 | .141 | .482 | .628 |
