# Supplementary material for: Suicidal ideation, plan, and attempt among men who have sex with men in Nepal: Findings from a cross-sectional study
Source: PLOS Glob Public Health. 2023 Nov 22;3(11):e0002348. doi: 10.1371/journal.pgph.0002348 (PMC10664887; doi:10.1371/journal.pgph.0002348)
Supplement: S2 Table — (DOCX) [file pgph.0002348.s005.docx]

**S2 Table:** Distribution of area under the curve of the suicidal plan with independent variables

| **Test Result Variable(s)** | **Area** | **Std. Error^a^** | **Asymptotic Sig.^b^** | **Asymptotic 95% Confidence Interval** | |
| --- | --- | --- | --- | --- | --- |
|  |  |  |  | **Lower Bound** | **Upper Bound** |
| Educational status | .591 | .038 | .017 | .516 | .666 |
| Age | .403 | .038 | .012 | .328 | .479 |
| Sexual orientation | .410 | .038 | .017 | .335 | .484 |
| Ever detained by police | .572 | .040 | .071 | .494 | .651 |
| Last time doctor’s visit | .415 | .037 | .023 | .342 | .488 |
| Food security | .586 | .040 | .031 | .508 | .664 |
| Violence | .554 | .040 | .183 | .475 | .633 |
| Depressive symptoms | .609 | .040 | .007 | .530 | .688 |
| Daytime sleepiness | .546 | .040 | .252 | .467 | .625 |
| Disclosed sexual orientation to anyone | .448 | .038 | .175 | .374 | .523 |
| Ever tested HIV | .607 | .037 | .004 | .534 | .680 |
