# Supplementary material for: Suicidal ideation, plan, and attempt among men who have sex with men in Nepal: Findings from a cross-sectional study
Source: PLOS Glob Public Health. 2023 Nov 22;3(11):e0002348. doi: 10.1371/journal.pgph.0002348 (PMC10664887; doi:10.1371/journal.pgph.0002348)
Supplement: S3 Table — (DOCX) [file pgph.0002348.s006.docx]

**S3 Table:** Distribution of area under the curve of the suicidal attempt with independent variables

| **Test Result Variable(s)** | **Area** | **Std. Error^a^** | **Asymptotic Sig.^b^** | **Asymptotic 95% Confidence Interval** | |
| --- | --- | --- | --- | --- | --- |
|  |  |  |  | **Lower Bound** | **Upper Bound** |
| Age | .399 | .043 | .018 | .315 | .483 |
| Education | .579 | .043 | .066 | .495 | .663 |
| Ever detained by police | .556 | .046 | .217 | .467 | .646 |
| Ever tested HIV | .587 | .042 | .038 | .505 | .669 |
| Depressive symptoms | .576 | .046 | .099 | .486 | .666 |
| Food security | .562 | .045 | .171 | .473 | .650 |
| Last time doctor’s visit | .415 | .040 | .036 | .336 | .495 |
