# Supplementary material for: Suicidal ideation, plan, and attempt among men who have sex with men in Nepal: Findings from a cross-sectional study
Source: PLOS Glob Public Health. 2023 Nov 22;3(11):e0002348. doi: 10.1371/journal.pgph.0002348 (PMC10664887; doi:10.1371/journal.pgph.0002348)
Supplement: S1 File — (DOCX) [file pgph.0002348.s007.docx]

**Suicidal ideation, plan, and attempt among men who have sex with men in Nepal: Findings from a cross-sectional study**

**Manuscript ID no**: PGPH-D-23-00620

STROBE Statement—checklist of items that should be included in reports of observational studies

|  | Item No. | Recommendation | Page  No. | Relevant text from manuscript |
| --- | --- | --- | --- | --- |
| **Title and abstract** | 1 | (*a*) Indicate the study’s design with a commonly used term in the title or the abstract | 1 | Suicidal ideation, plan, and attempt among men who have sex with men in Nepal: Findings from a cross-sectional study. |
|  |  | (*b*) Provide in the abstract an informative and balanced summary of what was done and what was found | 2,3 | A cross-sectional survey was conducted on 250 MSM between October and December 2022. Respondent-driven sampling method was used to recruit participants in this study. A structured questionnaire was used to investigate participants' demographic, behavioral, health-related, and psychosocial characteristics. Overall, the lifetime prevalence of suicidal ideation, plans, and attempts among MSM in this study were 42.4%, 31.2%, and 21.6%, respectively. Of the participants who reported experiencing suicidal ideation at some point in their lifetime (n=106), 22.6% thought about it multiple times in past 12 months. MSM with depressive symptoms (aOR = 5.7, 95% CI = 2.4-14.1), advanced education (higher secondary and above; aOR = 2.9, 95% CI = 1.4-6.1), and smoking habit (aOR = 2.5, 95% CI = 1.2-5.3) were at increased risk for suicidal ideation. |
| Introduction | | | |  |
| Background/rationale | 2 | Explain the scientific background and rationale for the investigation being reported | 5 | Despite the growing concerns about suicidal behaviors among MSM globally [12], much of this research has been conducted in high-income country settings [3, 5, 13, 14]. The extent to which these findings apply to SGM populations in LMIC settings, like Nepal, remains unknown. Particularly in Nepali culture, traditional gender roles significantly shape societal and familial expectations towards opposite-sex marriage and reproduction. As a result, MSMs who do not conform to these norms may be stigmatized and excluded by their families [15], contributing to an increased risk of suicide. Despite the growing number of suicides among MSM in Nepal [12], there is limited information regarding suicidal thoughts and conduct. So, there is a substantial need to scale research on mental health and suicidality among this population |
| Objectives | 3 | State specific objectives, including any prespecified hypotheses | 5 | Aimed to examine the prevalence of life time suicide ideation, plan, and attempts, and associated factors among MSM in Nepal, a low-income country |
| Methods | | | |  |
| Study design | 4 | Present key elements of study design early in the paper | 5 | Data were drawn from a cross-sectional study of 250 MSM. |
| Setting | 5 | Describe the setting, locations, and relevant dates, including periods of recruitment, exposure, follow-up, and data collection | 5,6 | Data were drawn from a cross-sectional study of 250 MSM conducted between October and December 2022 in Kathmandu Valley. The Kathmandu Valley is comprised of three districts: Kathmandu, Bhaktapur, and Lalitpur. Kathmandu district is the national capital, densely populated, and the largest metropolitan city whereas Bhaktapur, Lalitpur are neighboring districts inside the valley. |
| Participants | 6 | (*a*) *Cohort study*—Give the eligibility criteria, and the sources and methods of selection of participants. Describe methods of follow-up  *Case-control study*—Give the eligibility criteria, and the sources and methods of case ascertainment and control selection. Give the rationale for the choice of cases and controls  *Cross-sectional study*—Give the eligibility criteria, and the sources and methods of selection of participants | 6 | . Individuals were eligible if they identified as MSM; were aged 18 years or older; understood Nepali or English; and were willing to undergo screening for HIV and Syphilis. |
|  |  | (*b*) *Cohort study*—For matched studies, give matching criteria and number of exposed and unexposed  *Case-control study*—For matched studies, give matching criteria and the number of controls per case |  | NA |
| Variables | 7 | Clearly define all outcomes, exposures, predictors, potential confounders, and effect modifiers. Give diagnostic criteria, if applicable |  | NA |
| Data sources/ measurement | 8* | For each variable of interest, give sources of data and details of methods of assessment (measurement). Describe comparability of assessment methods if there is more than one group | *7* | The dependent variables in the study included suicidal behaviors. Suicidal behaviors were assessed using the suicidality module of the World Mental Health Composite International Diagnostic Interview (WHM-CIDI), a commonly used tool for measuring suicidal ideation, plan, and thoughts in different settings in many countries, including Nepal. This module includes an assessment of lifetime occurrences of suicide ideation (“*Have you ever thought about committing suicide?*”), plans (“*Have you ever made a plan for committing suicide?*”), and attempts (“*Have you ever attempted suicide?*”) |
| Bias | 9 | Describe any efforts to address potential sources of bias | NA | NA |
| Study size | 10 | Explain how the study size was arrived at | 6 | Respondent-driven sampling, a sampling technique based on social networks and typically employed for populations that are difficult to access, was used to recruit study participants [16]. We initiated the recruitment chains with five MSM “seeds”, purposively selected based on recommendations from community-based organizations working with MSM, with attention given to socio-demographic and geographic representation. Each seed who completed the interviewer-administered questionnaire was given five recruitment coupons to recruit potential peers. Each successive participant was provided with five coupons to enlist more peers in the study. All participants were required to give informed consent before commencing any study-related activities. |

Continued on next page

| Quantitative variables | 11 | Explain how quantitative variables were handled in the analyses. If applicable, describe which groupings were chosen and why |  | NA |
| --- | --- | --- | --- | --- |
| Statistical methods | 12 | (*a*) Describe all statistical methods, including those used to control for confounding | 10 | The statistical software IBM SPSS Version 26.0 (IBM Corp, New York, USA) was used for data analysis. Descriptive statistics were used to summarize the data, including frequency and percentages for categorical variables, and mean and standard deviation for continuous variables. The Chi-square test was used to assess the relationship between categorical independent and dependent variables. Multivariable logistic regression analysis was conducted to identify potential factors associated with the outcome variable. The adjusted odds ratio (AOR) was calculated with a 95% confidence interval (CI), and a p-value below 0.05 was statistically significant. Bivariate analysis was used to identify significant variables for inclusion in the adjusted regression analysis, with a 10% significance level used as the criterion for inclusion |
|  |  | (*b*) Describe any methods used to examine subgroups and interactions |  | NA |
|  |  | (*c*) Explain how missing data were addressed |  | NA |
|  |  | (*d*) *Cohort study*—If applicable, explain how loss to follow-up was addressed  *Case-control study*—If applicable, explain how matching of cases and controls was addressed.  *Cross-sectional study*—If applicable, describe analytical methods taking account of sampling strategy |  | NA |
|  |  | (*e*) Describe any sensitivity analyses |  | NA |
| Results | | | | |
| Participants | 13* | (a) Report numbers of individuals at each stage of study—eg numbers potentially eligible, examined for eligibility, confirmed eligible, included in the study, completing follow-up, and analysed |  | NA |
|  |  | (b) Give reasons for non-participation at each stage |  | NA |
|  |  | (c) Consider use of a flow diagram |  | NA |
| Descriptive data | 14* | (a) Give characteristics of study participants (eg demographic, clinical, social) and information on exposures and potential confounders |  | NA |
|  |  | (b) Indicate number of participants with missing data for each variable of interest |  | NA |
|  |  | (c) *Cohort study*—Summarise follow-up time (eg, average and total amount) |  | NA |
| Outcome data | 15* | *Cohort study*—Report numbers of outcome events or summary measures over time |  | *NA* |
|  |  | *Case-control study—*Report numbers in each exposure category, or summary measures of exposure |  | *NA* |
|  |  | *Cross-sectional study—*Report numbers of outcome events or summary measures | *11* | The lifetime prevalence of suicidal behaviors among study participants. Specifically, 42.4% of the participants reported suicidal ideation, 31.2% reported plans, and 21.6% reported attempts of suicide. Among the participants who had thoughts about ending their life in the past 12 months (n=106), 22.6% thought about it multiple times. |
| Main results | 16 | (*a*) Give unadjusted estimates and, if applicable, confounder-adjusted estimates and their precision (eg, 95% confidence interval). Make clear which confounders were adjusted for and why they were included | 33,34,35,36 | All the confounding variables controlled were shown in table 3 4, and 5. |
|  |  | (*b*) Report category boundaries when continuous variables were categorized | 7,8,9,10 | Age was dichotomized to *<25 years* and *≥25 years,* participants were asked to report their monthly income in Nepalese rupees (NPR), and this was reported with the categories: *<Nrs.20,000 (~ USD 150)* and *≥20,000 and above,* A composite score of 0-27 was calculated, and a score of >10 was regarded as having depressive symptoms, The ESS score (range: 0 to 24) was categorized as Normal (ESS score 0-10) and excessive daytime sleepiness symptoms (ESS score 11-24), Total scores for food insecurity responses, which ranged from 0 to 8, were added together. And the final scores were classified into 2 categories: food secure (0–3); and severe/moderately food insecure (4–8), The three-item scale of Oslo Social support had sum scores ranging from 3 to 14, and that score was operationalized into three categories of: Poor (3-8); Moderate (9-11); and Strong (12-14) |
|  |  | (*c*) If relevant, consider translating estimates of relative risk into absolute risk for a meaningful time period |  | NA |

Continued on next page

| Other analyses | 17 | Report other analyses done—eg analyses of subgroups and interactions, and sensitivity analyses |  | NA |
| --- | --- | --- | --- | --- |
| Discussion | | | | |
| Key results | 18 | Summarise key results with reference to study objectives | 12 | The lifetime prevalence of suicidal ideation (42.4%), plans (31.2%), and attempts (21.6%) among Nepalese MSM were alarmingly high compared to those from previous studies conducted in Nepal [12, 24] and other geographic settings [5, 13, 25]. Not surprisingly, these rates among MSM were higher than among Nepal's general population i.e. 13.3% suicidal ideation and 9.4% attempt [2, 26]. It is likely that discrimination, prejudice, stigma, and mental health issues brought about by their sexual orientation may have been attributed to the increased prevalence |
| Limitations | 19 | Discuss limitations of the study, considering sources of potential bias or imprecision. Discuss both direction and magnitude of any potential bias | 16 | Limitation is the potential for respondent bias, as the findings were based on subjective reports from MSM participants. Additionally, the study was conducted among MSM living in the Kathmandu valley, the capital city, so the results cannot be generalizable to MSM in other parts of Nepal. |
| Interpretation | 20 | Give a cautious overall interpretation of results considering objectives, limitations, multiplicity of analyses, results from similar studies, and other relevant evidence | 16 | The first limitation is that the study was conducted during the COVID-19 pandemic, so pandemic-related conditions might still influence suicidal behaviors. A longitudinal study design would be necessary to fully understand the pandemic's impact on mental health outcomes. Given the nature of the cross-sectional design, the results should be interpreted solely as associations, and the role of causality may not be inferred. |
| Generalisability | 21 | Discuss the generalisability (external validity) of the study results | 7 | Suicidal behaviors were assessed using the suicidality module of the World Mental Health Composite International Diagnostic Interview (WHM-CIDI), a commonly used tool for measuring suicidal ideation, plan, and thoughts in different settings in many countries, including Nepal. |
| Other information | |  | | |
| Funding | 22 | Give the source of funding and the role of the funders for the present study and, if applicable, for the original study on which the present article is based |  | There is no funding for this particular study solely. |

*Give information separately for cases and controls in case-control studies and, if applicable, for exposed and unexposed groups in cohort and cross-sectional studies.

**Note:** An Explanation and Elaboration article discusses each checklist item and gives methodological background and published examples of transparent reporting. The STROBE checklist is best used in conjunction with this article (freely available on the Web sites of PLoS Medicine at http://www.plosmedicine.org/, Annals of Internal Medicine at http://www.annals.org/, and Epidemiology at http://www.epidem.com/). Information on the STROBE Initiative is available at www.strobe-statement.org.
