# Supplementary material for: Suicidal ideation, plan, and attempt among men who have sex with men in Nepal: Findings from a cross-sectional study
Source: PLOS Glob Public Health. 2023 Nov 22;3(11):e0002348. doi: 10.1371/journal.pgph.0002348 (PMC10664887; doi:10.1371/journal.pgph.0002348)
Supplement: Attachment — Submitted filename: Response to Reviewers.docx [file pgph.0002348.s008.docx]

**Editorial Comments:**

1. Please send a completed 'Competing Interests' statement, including any COIs declared by your co-authors. If you have no competing interests to declare, please state "The authors have declared that no competing interests exist". Otherwise please declare all competing interests beginning with the statement "I have read the journal's policy and the authors of this manuscript have the following competing interests:"
   **Response**: Thank you, and we apologize for this oversight. We have added the following statement to address this comment:

“The authors have declared that no competing interest exists.”

2. We have noticed that you have stated in your data availability statement that raw data in the form of tables has been uploaded as supporting information. However, there are no corresponding files uploaded to the submission. Please upload them as separate files with the item type 'Supporting Information'.

**Response**: Thank you for the suggestion. As recommended, we have now uploaded the associated data to a public data repository and included the following statement:

“The raw data used for the analysis of this study have been deposited in the public data repository platform “Fig share” and can be easily accessed using the link 10.6084/m9.figshare.23750913.”

3. It is important to report the reliability and validity of the tools used in the study, along with justification for their selection.

**Response**: We agree and thank the reviewer for this suggestion. We have now reported the reliability and validity of tools used in the study within the Methods section.

4. The manuscript does not adhere to the STOBE guidelines, and it would be helpful to clearly state the inclusion and exclusion criteria in the methodology.

**Response**: We apologize and thank you for the suggestion. We have now included the inclusion and exclusion criteria within the Methods section to address this comment:

“Inclusion criteria: Individuals were eligible if they identified as MSM; were aged 18 years or older; understood Nepali or English; and were willing to undergo screening for HIV and Syphilis.

Exclusion criteria: The study excluded participants who did not come through referrals from previous study participants and those who did not identify as MSM, transgender males, or were below 18 years of age.”

5. Details regarding the fitting of the logistic regression model, assessment of assumptions, confounding, effect modification, and multicollinearity are lacking. Post-estimation tests such as sensitivity analysis, BIC, AIC, and VIF to check for collinearity, as well as reporting the ROC curve with AUC, should be included to ensure model fitness and predictive accuracy.

**Response**: Thank you for the suggestion. Now, we have made some edits within the statistical analysis section to address this comment:

“We tested for multi-collinearity by calculating variance inflation factor (VIF scores) for each variable in the predictor’s models. We set the cutoff VIF score of 5, and no variable was found to have a higher VIF score greater than 5. The model with the lowest Akaike Information Criterion (AIC) value was selected as the best-fitted model. The likelihood ratio and Hosmer Lemeshow tests were for the goodness of fit of the model.

The regression model was explained by the equation:

Log [Y/ (1-Y)] = b_0_ + b_1_ X_1_ + b_2_ X_2_ + b_3_ X_3_… ..b_n_X_n_ + e

Where Y is the expected probability for the outcome variable to occur, b_0_ is the constant/intercept, b_1_ through bn are the regression coefficients, X_1_ through X_n_ are distinct independent variables, and e is the error term.”

About the ROC curve: Our study was not aimed at validating these measures, therefore, did not include gold standard tools to compare. We are, therefore, unable to conduct any sensitivity and specificity analyses and generate ROC curves. Additionally, we would like to note that the measures used in this study have been validated and used in various settings and populations, including Nepal.

**Reviewer #1:**

1. “Were willing to undergo screening for HIV and Syphilis.” What was the reason for including this criterion?

**Response**: We apologize for not being clear in our original submission. The parent study was a biobehavioral survey, therefore, only those who indicated a willingness to undergo screening for HIV and syphilis were enrolled.

2. “Respondent-driven sampling” can also be stated as a non-random sampling technique.

**Response**: Thank you for the suggestion. We have now incorporated the suggested suggestion.

3. Report all reliability and validity of the tools used in this study. Justify why only these scales were used.

**Response**: We agree and thank the reviewer for this suggestion. We have now reported the reliability and validity of tools used in the study within the Methods section.

4. The reporting guidelines did not follow the STOBE guidelines. The inclusion and exclusion needed to be clearly stated in the methodology.

**Response**: We apologize and thank you for the suggestion. We have now included the inclusion and exclusion criteria within the Methods section to address this comment:

“Inclusion criteria: Individuals were eligible if they identified as MSM; were aged 18 years or older; understood Nepali or English; and were willing to undergo screening for HIV and Syphilis.

Exclusion criteria: The study excluded participants who did not come through referrals from previous study participants and those who did not identify as MSM, transgender males, or were below 18 years of age.”

5. Did Yates' continuity correction use in the Chi-square test?

**Response**: Thank you. We did not use the Yates’ continuity correction because none of the cells had an expected count smaller than 5. Yates’ correction may tend to overcorrect, which is unnecessary even with relatively low sample sizes too.

6. How did the logistic regression fit? How were the variables selected? Did the assumptions of the model fit? Did they check for confounding? Effect modification? Multi-collinearity? After fitting the model, did they check the model fitness, such as sensitivity analysis, BIC, and AIC? Post-estimation test, VIF requires to do to check for collinearity.

**Response**: Thank you for the suggestion. Now, we have made some edits within the statistical analysis section to address this comment:

“We tested for multi-collinearity by calculating variance inflation factor (VIF scores) for each variable in the predictor’s models. We set the cutoff VIF score of 5, and no variable was found to have a higher VIF score greater than 5. The model with the lowest Akaike Information Criterion (AIC) value was selected as the best-fitted model. The likelihood ratio and Hosmer Lemeshow tests were for the goodness of fit of the model.

The regression model was explained by the equation:

Log [Y/ (1-Y)] = b_0_ + b_1_ X_1_ + b_2_ X_2_ + b_3_ X_3_… ..b_n_X_n_ + e

Where Y is the expected probability for the outcome variable to occur, b_0_ is the constant/intercept, b_1_ through bn are the regression coefficients, X_1_ through X_n_ are distinct independent variables, and e is the error term.”

7. The ROC curve with the AUC should be reported to ensure that the models predicted the outcome.

**Response**: Our study was not aimed at validating these measures, therefore, did not include gold standard tools to compare. We are, therefore, unable to conduct any sensitivity and specificity analyses and generate ROC curves. Additionally, we would like to note that the measures used in this study have been validated and used in various settings and populations, including Nepal.

**Reviewer #2:**

1. I think that this is valuable information to add to the evidence base, and should be published *if* the authors are able to address an important methodological issue: in the respondent driven sampling paradigm that the authors use to sample this population, it seems quite plausible that there is important correlation between how many links an individual has in their social network and their experience with suicidal ideation, plans, and attempts. If social isolation is a risk factor for suicide, then the individuals who will have higher prevalence of suicidal ideation, plans, and attempts are precisely the individuals who are less likely to be included by RDS. I would like the authors to substantially expand their RDS methods, perhaps following the guidance suggested by Gile, Johnston, and Salganik in “Diagnostics for Respondent-driven Sampling,” Journal of the Royal Statistical Society Series A, 178:1, 241-269 (2015). (Some of the methods they prefer are implemented in their R package RDS <https://rdrr.io/cran/RDS/>)

   It is possible that refined methods for analyzing RDS data will lead to important changes to the substantive findings of this paper, and if so, I would expect them to be find that the disparities are even more stark.

**Response**: Thank you for this important suggestion. We have taken the opportunity to incorporate a network diagram into the method section, enhancing our study's clarity. Additionally, we implemented the refined methods for analyzing RDS data as suggested by the reviewer. However, the results were not significantly different.

In this study, we decided to incorporate analytical plans from the original submission to enable meaningful comparison with existing literature that has adopted a similar approach.

<https://journals.sagepub.com/doi/10.1177/0020764016663969>

<https://bmcpsychiatry.biomedcentral.com/articles/10.1186/s12888-016-0830-9>

<https://bmcpsychiatry.biomedcentral.com/articles/10.1186/s12888-021-03477-8>”

2. I think that the discussion (and introduction) could do more to quantitatively compare the prevalence of these suicidality indicators with the non-MSM (or total) population; I suggest even including it in your Figure 1 to make sure that busy readers cannot miss it.

**Response:** Thank you for the suggestion. We have now made some edits to the discussion and introduction sections to address this comment. However, as suggested, we could not include a figure since no study was done in the general population assessing suicidal ideation thoughts, plans, and attempts within a single paper. Additionally, the national-level prevalence of all three components currently does not exist.

3. Page 10: I would like to know if the cut-points for your dichotomous indicators were pre-planned or made in a data-driven way that you can describe.

**Response:** The cut of points for the dichotomous indicators were pre-planned as suggested by the previously published articles, which we cited in the methods sections.

4. Page 6 and 14: the term “interestingly” does not sound appropriately serious for this stark finding. I prefer how you said “Notably” on page 16.

**Response:** Thank you for the suggestions. We have now made the necessary edits to address this comment.
