# Supplementary material for: Suicidal ideation, plan, and attempt among men who have sex with men in Nepal: Findings from a cross-sectional study
Source: PLOS Glob Public Health. 2023 Nov 22;3(11):e0002348. doi: 10.1371/journal.pgph.0002348 (PMC10664887; doi:10.1371/journal.pgph.0002348)
Supplement: Attachment — Submitted filename: Rebuttal_Letter_PGPH_10.docx [file pgph.0002348.s009.docx]

October 18, 2023

Editor

PLOS Global Public Health

Re: PGPH-D-23-00620) - [EMID:7441a38815b9a12c] *“Suicidal ideation, plan, and attempt among men who have sex with men in Nepal: Findings from a cross-sectional study”*

Dear Rakesh Singh,

Thank you so much for your and reviewer’s comments on our manuscript entitled, "Suicidal ideation, plan, and attempt among men who have sex with men in Nepal: Findings from a cross-sectional study”. We have carefully revised the manuscript in response to the extensive and insightful comments we received. We are pleased that there is interest in the topic and are grateful for constructive comments.

We have addressed the comments point by point and have made corresponding changes in the manuscript in track changes (see below).

We hope this revised version will be suitable for publication in PLOS Global Public Health.

Sincerely,

Dr. Roman Shrestha, Ph.D., MPH

University of Connecticut

**Journal Requirements:**

**1. Please amend your detailed Financial Disclosure statement. This is published with the article. It must therefore be completed in full sentences and contain the exact wording you wish to be published.**

a. State the initials, alongside each funding source, of each author to receive each grant.
b. State what role the funders took in the study. If the funders had no role in your study, please state: “The funders had no role in study design, data collection and analysis, decision to publish, or preparation of the manuscript.”
c. If any authors received a salary from any of your funders, please state which authors and which funders.

If you did not receive any funding for this study, please simply state: “The authors received no specific funding for this work.”

Thank you for your suggestions. Now we completed the full sentence and contain the exact wording as we wish to be published in the manuscript and the submission portal.

**Reviewer #1: “How did the logistic regression fit? How were the variables selected? Did the assumptions of the model fit? Did they check for confounding? Effect modification?” these comments were not addressed. Authors cannot select all variables from the model of univariate analysis. Because the non-associated variable in the adjusted model may reduce the predictive accuracy. “After fitting the model, did they check the model fitness, such as sensitivity analysis, BIC, and AIC?” authors can do model fitness test that authors have already done in authors’ adjusted model. The ROC curve with the AUC should be reported to ensure that the models predicted the outcome.” For AUC and ROC, authors do not need separate study or tool validation. Authors’ logistic regression model should give AUC and ROC that will help authors to know the predictive accuracy of the model.**

We thank the reviewer for the valuable suggestions. We agree with the reviewer's observation that the incorporation of non-associated variables into the adjusted model could potentially diminish predictive accuracy. As detailed in the Methods section, we intentionally refrained from including all variables in our analysis. Initially, we conducted a bivariate analysis to identify significant variables suitable for inclusion in the adjusted regression analysis, employing a 10% significance level as the inclusion criterion. Furthermore, we assessed multicollinearity by computing the variance inflation factor (VIF) score for each predictor variable. We established a VIF score threshold of 5, and no variable exceeded this threshold. To determine the most appropriate model, we selected the one with the lowest Akaike Information Criterion (AIC) value. Additionally, we employed likelihood ratio and Hosmer-Lemeshow tests to assess the model's goodness of fit.

In response to the reviewer’s recommendation, we have now incorporated AUC and ROC analyses into our logistic regression model. The AUC value, which ranges from 0 to 1, quantifies the model ability to distinguish between different classes. We have also made some edits within the Discussion section to indicating some limitations of our analytical plan.
